# Tubby-like protein superfamily member PLSCR3 functions as a negative regulator of adipogenesis in mouse 3T3-L1 preadipocytes by suppressing induction of late differentiation stage transcription factors

**DOI:** 10.1042/BSR20150215

**Published:** 2016-01-22

**Authors:** Akira Inokawa, Tatsutoshi Inuzuka, Terunao Takahara, Hideki Shibata, Masatoshi Maki

**Affiliations:** * Department of Applied Molecular Biosciences, Graduate School of Bioagricultural Sciences, Nagoya University, Furo-cho, Chikusa-ku, Nagoya 464-8601, Japan

**Keywords:** 3T3-L1, adipogenesis, extracellular microvesicles, negative regulator, transcription factors, unconventional secretion

## Abstract

PLSCR3 (phospholipid scramblase 3, Scr3) belongs to the superfamily of membrane-associated transcription regulators named Tubby-like proteins (TULPs). Physiological phospholipid scrambling activities of PLSCRs *in vivo* have been skeptically argued, and knowledge of the biological functions of Scr3 is limited. We investigated the expression of Scr3 during differentiation of mouse 3T3-L1 preadipocytes by Western blotting (WB) and by reverse-transcription and real-time quantitative PCR (RT-qPCR). The Scr3 protein decreased during 3T3-L1 differentiation accompanied by a reduction in the mRNA level, and there was a significant increase in the amount of Scr3 protein secreted into the culture medium in the form of extracellular microvesicles (exosomes). On the other hand, Scr3 expression did not significantly decrease, and the secretion of Scr3 in 3T3 Swiss-albino fibroblasts (a parental cell-line of 3T3-L1) was not increased by differentiation treatment. Overexpression of human Scr3 during 3T3-L1 differentiation suppressed triacylglycerol accumulation and inhibited induction of the mRNAs of late stage pro-adipogenic transcription factors [CCAAT/enhancer-binding protein α (C/EBPα) and peroxisome proliferator-activated receptor γ (PPARγ)] and X-box-binding protein 1 (XBP1). Expression of early stage pro-adipogenic transcription factors (C/EBPβ and C/EBPδ) was not significantly affected. These results suggest that Scr3 functions as a negative regulator of adipogenesis in 3T3-L1 cells at a specific differentiation stage and that decrease in the intracellular amount of Scr3 protein caused by reduction in Scr3 mRNA expression and enhanced secretion of Scr3 protein appears to be important for appropriate adipocyte differentiation.

## Abstract (precis)

Decrease in intracellular amount of phospholipid scramblase 3 (PLSCR3) is accompanied by enhanced unconventional secretion during differentiation of mouse preadipocytic 3T3-L1 cells. Forced overexpression of PLSCR3 in 3T3-L1 cells inhibited adipogenesis by suppressing induction of late stage pro-adipogenic transcription factors.

## INTRODUCTION

PLSCR1 (phospholipid scramblase 1, Scr1), first isolated from human erythrocytes, was reported to be a factor catalysing movement of phospholipids bi-directionally between outer and inner leaflets (scrambling) to disrupt the asymmetrical phospholipid distribution in a Ca^2+^-dependent manner [1]. Five PLSCR paralogues (*PLSCR1–5*) are found in the mammalian genomes. Despite a number of reports describing Ca^2+^-dependent phospholipid scrambling activities *in vitro* [1–6], the idea of PLSCRs as physiological phospholipid-translocating proteins working *in vivo* has been skeptically argued [7]. *Plscr1^−/−^* mice showed no haemostatic defects and showed normal phosphatidylserine exposure upon activation [8]. Moreover, genuine plasma membrane-integrated scrambling factors (TMEM16F and Xkr8) have been identified by cell-based *in vivo* assays of phosphatidylserine exposure activities [9–11].

In earlier studies, PLSCRs were predicted to have a respective C-terminal transmembrane helix [2,7,12]. The results of a recent bioinformatics study [13], however, suggested that PLSCRs have globular domains similar to the C-terminal domains of membrane-tethered transcription factors named Tubby (TUB) and Tubby-like proteins (TULPs), which have 12-stranded β barrels filled with C-terminal hydrophobic helices in the centre [14,15]. TUB and TULPs bind phosphatidylinositol 4,5-bisphosphate (PIP_2_) and are liberated from the plasma membrane upon activation of G-protein-coupled receptors [15,16]. It is still not clear whether the topology of the C-terminal α-helical regions of PLSCRs is a transmembrane helix [4,17] or a helix that fills the central hole of the β-barrel [13,18]. However, PLSCRs have been shown to contain functional non-classical nuclear localization signals [19,20], and thus the nuclear translocation of PLSCRs by treatment with a palmitoylation inhibitor (2-bromo-palmitate) or by palmitoylation site mutation favour the latter hypothesis at least *in vivo* [18,21].

Among the five mammalian PLSCR isoforms (PLSCR1-5), Scr1 (PLSCR1) has been most extensively studied, and functions apparently unrelated to phospholipid scrambling activities have been suggested: (i) cell signalling by interacting with cell surface receptors and a subset of Src-family kinases [22–25] and (ii) transcriptional regulation in haematopoietic cell differentiation [26,27]. Consistent with the structural similarity to TUB and TULPs, Scr1 has been shown to directly bind to the inositol 1,4,5-trisphosphate receptor 1 (IP_3_R1) promoter region and enhance its expression [27,28]. However, gene expression regulatory functions have not yet been reported for other PLSCRs. A few studies have suggested that Scr3 is phosphorylated by PKCδ and is involved in apoptosis in the mitochondrial pathway [3,29]. Disruption of the mouse Scr3 gene (*Plscr3*^−/−^) resulted in accumulation of abdominal fat, insulin resistance, glucose intolerance and dyslipidemia in adult mice [30], whereas Scr1 gene knockout mice (*Plscr1^−/−^*) showed no profound effects on adipogenesis but displayed prenatal granulocytopenia [8,30]. In the present study, to gain an insight into the function of Scr3 in adipogenesis, we investigated roles of Scr3 in differentiation of 3T3-L1 cells, which are commonly used preadipocytic cell-line cells derived from mouse 3T3 Swiss-albino fibroblasts [31–33]. We found that Scr3 is down-regulated during differentiation and that overexpression of human Scr3 (hScr3) attenuates the expression of late differentiation stage transcription factors and X-box-binding protein 1 (XBP1), a transcriptional mediator of the unfolded protein response (UPR) that is implicated in adipogenesis [34], leading to suppression of adipogenesis.

## MATERIALS AND METHODS

### Antibodies and reagents

Mouse monoclonal antibodies against glyceraldehyde-3-phosphate dehydrogenase (GAPDH) and flotillin-2 were obtained from Santa Cruz (clone 6C5) and from BD Transduction Laboratories respectively. Preparation of rabbit antiserum against GST-fused hScr3 N-terminal region (1–78 amino acids) protein was described previously [35]. In the present study, the anti-Scr3 antibodies were affinity-purified using immobilized maltose-binding protein (MBP)-fused Scr3, and residual antibodies reacting with GST were immuno-absorbed with GST-immobilized beads. Dexamethasone (Dex), 3-isobutyl-1-methylxanthine (IBMX) and thapsigargin (Tg), an irreversible inhibitor of sarcoplasmic/endoplasmic reticulum (ER) Ca^2+^-ATPase (SERCA), were purchased from Wako Pure Chemicals. Insulin was obtained from Sigma–Aldrich.

### Plasmid construction and retroviral infection

A 0.9-kbp fragment of previously cloned hScr3 cDNA [35] was inserted into the BamHI site of pCX4pur, a murine leukaemia virus-based retrovirus vector [36]. Platinum-E (PLAT-E) cells, retrovirus packaging cells expressing gag-pol and env (kindly provided by Dr Toshio Kitamura, University of Tokyo) [37], were transfected with an empty vector or pCX4pur/hScr3 using FuGENE®6 (Promega) to produce recombinant retroviruses. After the conditioned culture medium had been centrifuged to remove cell debris, the resultant supernatants were filtered through a 0.2-μm filter (Advantech) and used for infection of 3T3-L1 cells in the presence of 8 μg/ml of polybrene (hexadimethrine bromide, Sigma–Aldrich).

### Cell culture and induction of adipogenesis

3T3-L1 and 3T3 Swiss-albino cells were obtained from JCRB Cell Bank (JCRB9014 and JCRB9019) and maintained in a maintenance medium consisting of Dulbecco's modified Eagle medium (DMEM) (Nissui Pharmaceutical) supplemented with 4 mM glutamine, 10% FBS, 100 μg/ml streptomycin and 100 units/ml penicillin. After reaching confluency, culture was continued for further 2 days (designated day 0), and cells were incubated in a differentiation induction medium (maintenance medium supplemented with 1 μM Dex, 0.5 mM IBMX and 1.7 μM insulin) essentially as described previously [38,39]. On day 2, the medium was replaced with a differentiation medium (maintenance medium supplemented with 1.7 μM insulin), and the culture was continued until day 8 with medium change at 48-h intervals.

### Western blotting

Total cell lysates (TCLs) were obtained from cells that were lysed with a buffer (20 mM HEPES-KOH, pH 7.4, 142.5 mM KCl, 1.5 mM MgCl_2_) containing 0.2% Nonidet P-40 and protease inhibitors (3 μg/ml leupeptin, 0.1 mM pefabloc, 10 μM E-64, and 1 μM pepstatin), followed by sonication. To prepare CM-P_100_ fractions, the conditioned medium recovered from each 48-h culture was centrifuged at 10000 × ***g*** for 15 min at 4°C to remove aggregates and cell debris, and the supernatant was further centrifuged at 100000 × ***g*** (Beckman rotor TLA100.3, 46000 rev./min) for 1 h at 4°C. The pellets were solubilized in SDS/PAGE sample buffer and used as CM-P_100_ fractions. Proteins were resolved by SDS/PAGE, transferred to PVDF membranes (Immobilon®-P, Merck/Millipore), and incubated with primary antibodies followed by horseradish peroxidase (HRP)-conjugated secondary antibodies. Chemiluminescent signals were detected with a luminescent image analyser, LAS-3000mini (Fuji Film), using SuperSignal® West Pico Chemiluminescent Substrate (Thermo Fisher Scientific). Signals of bands on Western blotting (WB) were quantified by ImageJ software.

### RT-qPCR

Total RNA was extracted using Sepasol®-RNA I super G (Nacalai Tesque), and contaminating DNA was removed by digestion with DNase (Nippon Gene). Reverse-transcription and real-time quantitative PCR (RT-qPCR) were performed using a PrimeScript® RT reagent kit (Perfect Real Time, RR037A, Takara Bio) and FastStart Essential DNA Green Master (Roche Applied Science) respectively. Synthesized cDNA was analysed by LightCycler® Nano (Roche Applied Science) using specific primers shown in Table 1. Data were analysed according to the manufacturer's instructions.

**Table 1:** List of primers used for RT-qPCR *Primers for tXBP1 are designed for amplification of both unspliced XBP1 (uXBP1) mRNA and IRE1-dependent spliced XBP1 (sXBP1) mRNA in the cytoplasmically unspliced region.

	Primer sequences	
Transcripts	Forward	Reverse
Scr3	5′-tactgctgggagccacgtt-3′	5′-gcttctaactggtgatggcagag-3′
PPARγ	5′-ggagttcctcaaaagcctgcg-3′	5′-ttggatgtcctcgatgggct-3′
C/EBPα	5′-caagaacagcaacgagtaccg-3′	5′-aggcggtcattgtcactggt-3′
C/EBPβ	5′-agaagacggtggacaagctga-3′	5′-gtcagctccagcaccttgtg-3′
C/EBPδ	5′-ttctacgagccaggcagggt-3′	5′-gcggccatggagtcaatgta-3′
aP2	5′-tcaccatccggtcagagagta-3′	5′-ctttcataacacattccaccaccag-3′
18S rRNA	5′-ttgactcaacacgggaaacc-3′	5′-tcgctccaccaactaagaacg-3′
tXBP1*	5′-gtgcaggcccagttgtca-3′	5′-ggtccttctgggtagacctctg-3′
sXBP1	5′-ctgagtccgaatcaggtgcag-3′	5′-gtccatgggaagatgttctgg-3′

### Miscellaneous

Amounts of triacylglycerols and TCL proteins were measured with an Adipogenesis Assay Kit (BioVision) and Pierce BCA Protein Assay Kit (Thermo Fisher Scientific) respectively. Oil Red O staining was performed essentially as described previously [34]. Statistical analysis was performed by one-way analysis of variance (ANOVA) followed by Tukey's test for time-course data sets or by Student's *t* test for comparison of data between two cell lines or between two experimental conditions. The values (*P*<0.05) are considered statistically significant.

## RESULTS

### Expression of Scr3 in 3T3 Swiss-albino cells and 3T3-L1 cells

**Figure 1:**
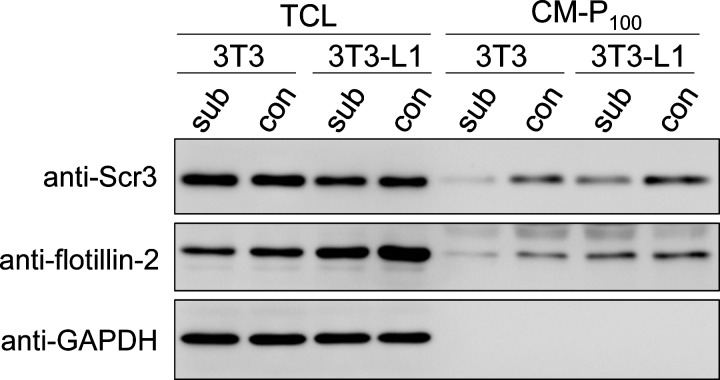
Expression of Scr3 protein in 3T3 cells and 3T3-L1 cells Mouse 3T3 cells and 3T3-L1 cells were grown to subconfluent (sub) and confluent (con) cell monolayers, and TCLs and 100000 × ***g*** pellets of the conditioned medium (CM-P_100_) were analysed by WB using specific antibodies as indicated. Equal amounts of proteins (each 9.7 μg) in TCLs were used for analysis, but equal aliquots of 100000 × ***g*** pellets from an equal volume of the conditioned medium were used for comparison of CM-P_100_.

We compared the expression levels of Scr3 in 3T3 Swiss-albino cells and preadipocytic 3T3-L1 cells by WB since 3T3-L1 is a subline of mouse 3T3 Swiss-albino fibroblasts [31,32]. As shown in Figure 1, intensity of the WB signal of Scr3 in the TCL was slightly stronger in 3T3 cells than in 3T3-L1 cells under both subconfluent (*sub*) and confluent (*con*) conditions despite weaker or similar signal intensities of the two loading controls used (*flotillin-2* and *GAPDH*). We previously found that Scr3, either exogenously expressed in human embryonic kidney (HEK)293 cells or endogenously expressed in human bladder carcinoma T-24 cells, was consistently secreted into the culture medium in the form of extracellular microvesicles (exosomes) [18]. Likewise, secretion of Scr3 as the form of exosomes was also observed in mouse 3T3 and 3T3-L1 cells (Figure 1). The amounts of Scr3 and flotillin-2 proteins in 100000 × ***g*** pellets from the 10000 × ***g*** supernatant of the conditioned medium (CM-P_100_ fraction) of 3T3-L1 cells were greater than those derived from 3T3 cells. On the other hand, the GAPDH protein was not detected in the CM-P_100_ fraction. Scr3 was recovered in fractions of the density of 1.10–1.13 g/cm^3^ by sucrose density gradient analysis of the CM-P_100_ fraction (results not shown), indicating that the Scr3-containing extracellular microvesicles are in accordance with the criteria of exosomes [40].

### Reduction of Scr3 expression in 3T3-L1 cells during differentiation

**Figure 2:**
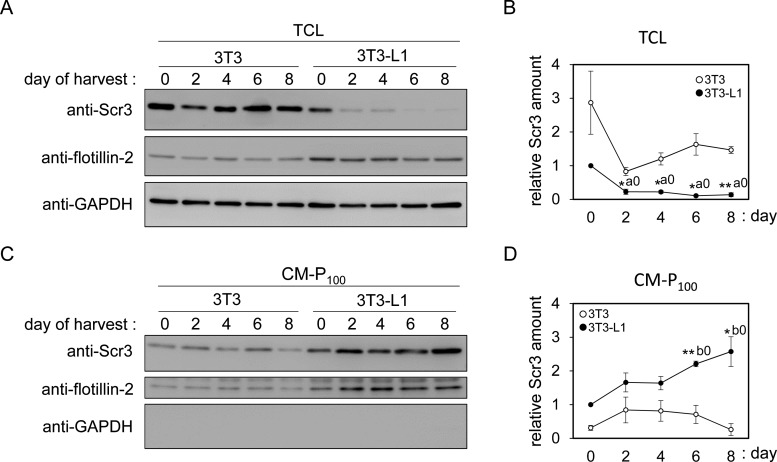
Differences in expression of Scr3 protein by adipogenic stimulation (**A**) TCLs of 3T3 and 3T3-L1 cells harvested on the indicated days and (**C**) 100000 × ***g*** pellets of the conditioned medium (CM-P_100_) were subjected to WB analysis using specific antibodies as indicated. Equal amounts of TCL proteins (5.1 μg each) and aliquots of CM-P_100_ fractions recovered from an equal volume of the conditioned medium were analysed. Representative data from three independent experiments are shown. WB signals were quantified, and changes in the relative amount of Scr3 protein compared with the amount on day 0 in 3T3-L1 cells (set to 1.0) are shown in graphs (mean ± S.E.M.; *n*=3) for TCL (**B**) and CM-P_100_ (**D**) of 3T3 cells (unfilled circles) and 3T3-L1 cells (filled circles). Statistical significance by Student's *t* test (*n*=3): *P<0.05; **P<0.01, for comparison between 3T3 and 3T3-L1 cells on each indicated day. Statistical significance by Tukey's test (*n*=3): a0 (*P*<0.001), days 2, 4, 6 and 8 compared with day 0 in 3T3-L1 cells; b0 (*P*<0.05), days 6 and 8 compared with day 0 in 3T3-L1 cells. Quantified data for GAPDH and flotillin-2 in 3T3-L1 cells are shown in Supplementary Figure S2.

**Figure 3:**
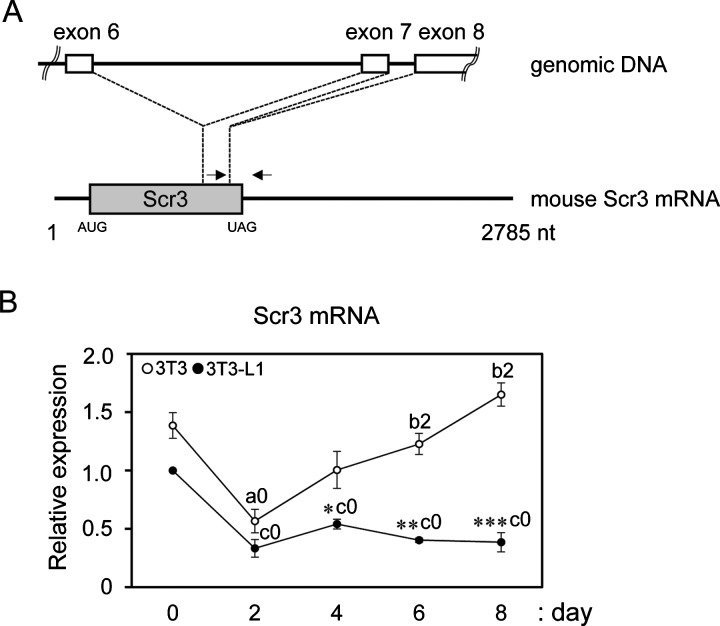
RT-qPCR analysis of Scr3 mRNA (**A**) Primers used for RT-qPCR are shown by arrows above the line indicating the mouse Scr3 mRNA (NCBI reference sequence: NM_023564.4). The sequences of forward and reverse primers are located in exon 7 and exon 8 respectively, in the mouse Scr3 gene and are compatible with human Scr3 mRNA. Location of the Scr3 protein coding region is shown by a grey closed box. (**B**) Total RNA was isolated from the cells on the indicated culture days, and RT-qPCR was performed as described in 'Materials and Methods'. The relative Scr3 mRNA quantity was calculated by the comparative Cq (quantitative cycles) method using 18S rRNA as a reference RNA. Relative expression in comparison with the value of 3T3-L1 cells on day 0 (set to 1.0) is presented (mean ± S.E.M.; *n*=3). Unfilled circles, 3T3 cells; filled circles, 3T3-L1 cells. Statistical significance by Student's *t* test (n=3): *P<0.05; **P<0.01; ***P<0.001, for comparison between 3T3 and 3T3-L1 cells on each indicated day. Statistical significance by Tukey's test (*n*=3): a0 (*P*<0.01), day 2 compared with day 0 in 3T3 cells; b2 (*P*<0.05), days 6 and 8 compared with day 2 in 3T3 cells; c0 (*P*<0.001), days 2, 4, 6 and 8 compared with day 0 in 3T3-L1 cells.

Adipogenesis of 3T3-L1 cells was induced by a standard protocol by addition of induction reagents (Dex, IBMX and insulin) to confluent cells on day 0 followed by replacement with a medium containing insulin every other day until day 8 as an experimental schedule shown in Supplementary Figure S1A. For comparison, 3T3 Swiss-albino cells were also treated with adipogenic reagents. Differentiation of 3T3-L1 cells, but not that of 3T3 cells, was confirmed by lipid staining with Oil-Red O (Supplementary Figure S1B). We investigated changes in Scr3 expression during adipogenic stimulation of those cells. The amount of Scr3 protein estimated by WB in TCL was significantly decreased on day 2, and the lower level was sustained thereafter until the last day of the assay (WB signal intensity of 13.9–22.6% compared with day 0) in 3T3-L1 cells, whereas the amount of Scr3 protein in 3T3 cells during stimulation was maintained at higher level than that of 3T3-L1 cells (Figure 2A; quantified data shown in Figure 2B). The amounts of control proteins analysed in this study decreased slightly (*Flotillin-2*) or remained constant (*GAPDH*) in both 3T3-L1 and 3T3 cells (quantified data for 3T3-L1 cells shown in Supplementary Figure S2A). Interestingly, Scr3 protein in the CM-P_100_ fraction was increased by adipogenic stimulation in 3T3-L1 cells (WB signal intensity of 257% on day 8) but was only marginally changed in 3T3 cells (Figure 2C; quantified data shown in Figure 2D). Flotillin-2 protein was not significantly elevated in either 3T3-L1 or 3T3 cells (quantified data for 3T3-L1 cells shown in Supplementary Figure S2B). Among the three induction reagents (Dex, IBMX and insulin), IBMX and insulin were found to reduce the expression of Scr3 protein on day 2 in 3T3-L1 cells (Supplementary Figure S3A; quantified data shown in Supplementary Figure S3B), whereas the amount of Scr3 protein in 3T3 cells was not significantly reduced by any combinations (Supplementary Figure S3C; quantified data shown in Supplementary Figure S3D). To investigate whether the Scr3 mRNA level also decreases during adipogenesis of 3T3-L1 cells, we performed RT-qPCR using 18S rRNA as a reference gene transcript. We designed a pair of RT-qPCR primers that correspond to sequences in separate exons in the Scr3 gene but give an 87-bp PCR product using the Scr3 cDNA as a template (Figure 3A). As shown in Figure 3B, the expression of Scr3 mRNA was temporarily suppressed on day 2 but was gradually recovered thereafter in 3T3 cells (unfilled circles). In contrast, the relative amount of Scr3 mRNA in 3T3-L1 cells (filled circles) was decreased to a lower level (33.2–54.0% compared with day 0) throughout the period.

### Effect of human Scr3 overexpression on adipocyte differentiation

**Figure 4:**
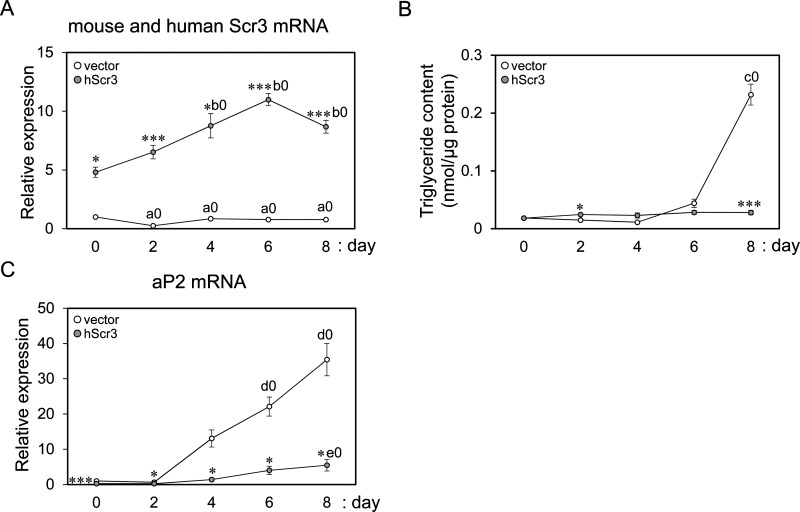
Suppression of adipogenesis in 3T3-L1 cells by overexpression of human Scr3 Effects of overexpression of hScr3 on adipogenesis were investigated by infecting 3T3-L1 preadipocytes with retroviruses expressing hScr3 or with control viruses (pCX4pur empty vector) in triplicate and inducing the infected cells for adipocyte differentiation (day 0) as similarly described in the legend to Supplementary Figure S1A. RT-qPCR was performed to quantify (**A**) the total amounts of expressed murine and human Scr3 (mouse and human Scr3) mRNAs using compatible Scr3 primers and (**C**) aP2 mRNA with specific primers shown in Table 1. Relative expression levels were calculated by comparison with the values of control cells on day 0 (set to 1.0), and 18S rRNA was used as a reference RNA for normalization. (**B**) Triacylglycerol contents in the cells harvested on indicated differentiation days were measured and expressed as nmol/μg protein. All data in (A), (B) and (C) are presented as mean ± S.E.M. (*n*=3). Unfilled circles, control cells; grey circles, hScr3 overexpressed cells. Statistical significance by Student's *t* test (*n*=3): *P<0.01; ***P<0.001, for comparison between control cells and hScr3-overexpressed cells on each indicated day. Statistical significance by Tukey's test (*n*=3): a0 (*P*<0.05), days 2, 4, 6 and 8 compared with day 0 in control cells; b0 (*P*<0.05), days 4, 6 and 8 compared with day 0 in hScr3-overexpressed cells; c0 (*P*<0.001), day 8 compared with day 0 in control cells; d0 (*P*<0.01), days 6 and 8 compared with day 0 in control cells; e0 (*P*<0.05), day 8 compared with day 0 in hScr3-overexpressed cells; *P*=0.052, day 4 compared with day 0 in control cells.

To understand the biological significance of the specific decrease in expression of Scr3 for 3T3-L1 adipocyte differentiation, we investigated effect of overexpression of hScr3 (93% amino acid identity with mouse Scr3, mScr3) by a retrovirus expression system on differentiation of 3T3-L1 cells. As shown in Figure 4A, RT-qPCR analysis using PCR primers common to both mouse and human Scr3 mRNAs revealed 5- to 11-fold overexpression of murine and human Scr3 mRNAs (*mouse* and *human Scr3 mRNA*) in the hScr3 retrovirus-infected cells (grey circles) compared with that in empty vector retrovirus-infected control cells (unfilled circles) throughout the differentiation period. Triacylglycerol contents increased significantly on day 8 in the control cells, but markedly reduced in the hScr3-overexpressed cells (Figure 4B). We also measured expression of the mRNA of aP2 (adipocyte protein 2; fatty acid-binding protein 4), a known marker of adipocyte differentiation [41]. As expected, expression of the aP2 mRNA increased in the later differentiation period in the control cells, but it was markedly reduced by overexpre-ssion of hScr3 (Figure 4C).

### Effects of hScr3 overexpression on an adipogenic transcriptional cascade

**Figure 5:**
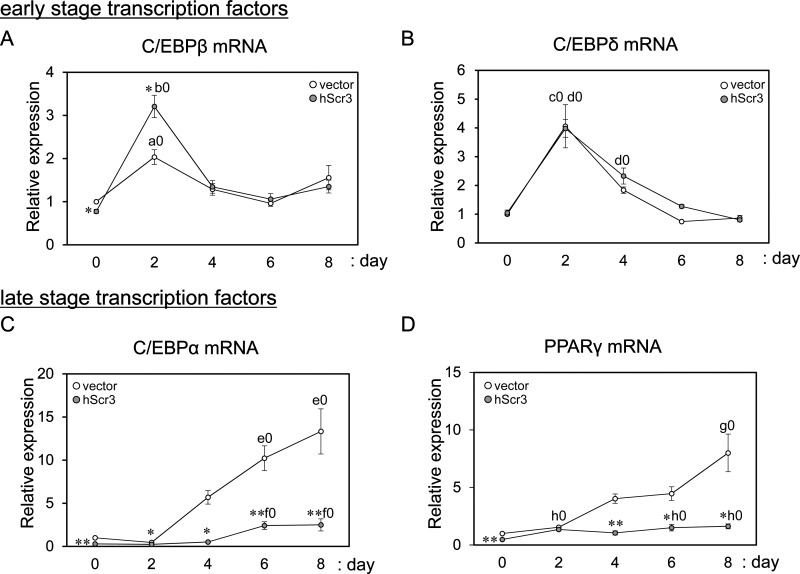
Effects of hScr3 overexpression on induction of pro-adipogenic transcription factors Quantification of mRNAs encoding early differentiation stage transcription factors, C/EBPβ (**A**) and C/EBPδ (**B**), and late differentiation stage transcription factors, C/EBPα (**C**) and PPARγ (**D**), was performed by RT-qPCR for control retrovirus-infected cells and hScr3-overexpressed cells that were harvested on indicated cultured days during adipogenic differentiation. Quantification of relative expression of each transcription factor mRNA (set to 1.0 in control vector expressed 3T3-L1 cells on day 0) was similarly performed as described in the legend to Figure 4. All data are presented as mean ± S.E.M. (*n*=3). Unfilled circles, control cells; grey circles, hScr3-overexpressed cells. Statistical significance by Student's *t* test (*n*=3): *P<0.05; **P<0.01, for comparison between control cells and hScr3-overexpressed cells on each indicated day*.* Statistical significance by Tukey's test (*n*=3): a0 (*P*<0.01), day 2 compared with day 0 in control cells; b0 (*P*<0.001), day 2 compared with day 0 in hScr3-overexpressed cells; c0 (*P*<0.001), day 2 compared with day 0 in control cells; d0 (*P*<0.01), days 2 and 4 compared with day 0 in hScr3-overexpressed cells; e0 (*P*<0.01), days 6 and 8 compared with day 0 in control cells; f0 (*P*<0.05), days 6 and 8 compared with day 0 in hScr3-overexpressed cells; g0 (*P*<0.001), day 8 compared with day 0 in control cells; h0 (*P*<0.01), days 2, 6 and 8 compared with day 0 in hScr3-overexpressed cells.

Adipocyte differentiation is regulated by extracellular signals and a cascade of transcription factors [42,43]. CCAAT-enhancer-binding protein β (C/EBPβ) and C/EBPδ are early differentiation stage transcription factors that are induced by IBMX (a phosphodiesterase inhibitor causing elevation of cyclic nucleotide concentrations) and Dex (a glucocorticoid analogue) respectively [44]. On the other hand, peroxisome proliferator-activated receptor γ (PPARγ) and C/EBPα, which are induced by C/EBPβ and C/EBPδ and are activated via the insulin pathway, induce and maintain each expression reciprocally and promote induction of adipocyte-specific genes such as *aP2* at the terminal stage of differentiation, leading to the adipocyte phenotype [45,46]. To know which stage of the adipocyte differentiation was affected by overexpression of hScr3, we measured expression levels of early stage pro-adipogenic transcription factors (C/EBPβ and C/EBPδ) and late stage pro-adipogenic transcription factors (PPARγ and C/EBPα) by RT-qPCR. Although hScr3 overexpression had no significant effects on the expression patterns of C/EBPβ and C/EBPδ mRNAs compared with those in empty vector control cells except for a slightly greater enhancement of C/EBPβ expression on day 2 (Figures 5A and 5B), it markedly suppressed the expression of C/EBPα and PPARγ mRNAs (Figures 5C and 5D).

### Effects on induction of XBP1 pre-mRNA and splicing

A line of evidence indicates that ER stress is linked with lipid metabolism disorders leading to obesity [47]. Three pathways involving ER-transmembrane stress sensory proteins (IRE1, PERK and ATF6) are known to respond to accumulation of misfolded or unfolded proteins in the ER (UPR) and antagonize experimentally evoked cellular stress [48]. Activated IRE1α triggers unconventional cytoplasmic splicing of XBP1 mRNA to generate the mature spliced form, the translation product of which (designated sXBP1) translocates to the nucleus and activates transcription of genes involved in protein folding (ER chaperones) and ER-associated degradation (ERAD) to restore ER homoeostasis. Recent studies have shown that expression of XBP1 is up-regulated in 3T3-L1 cells during differentiation and that the cytoplasmic splicing of XBP1 mRNA is enhanced [34,38].

**Figure 6:**
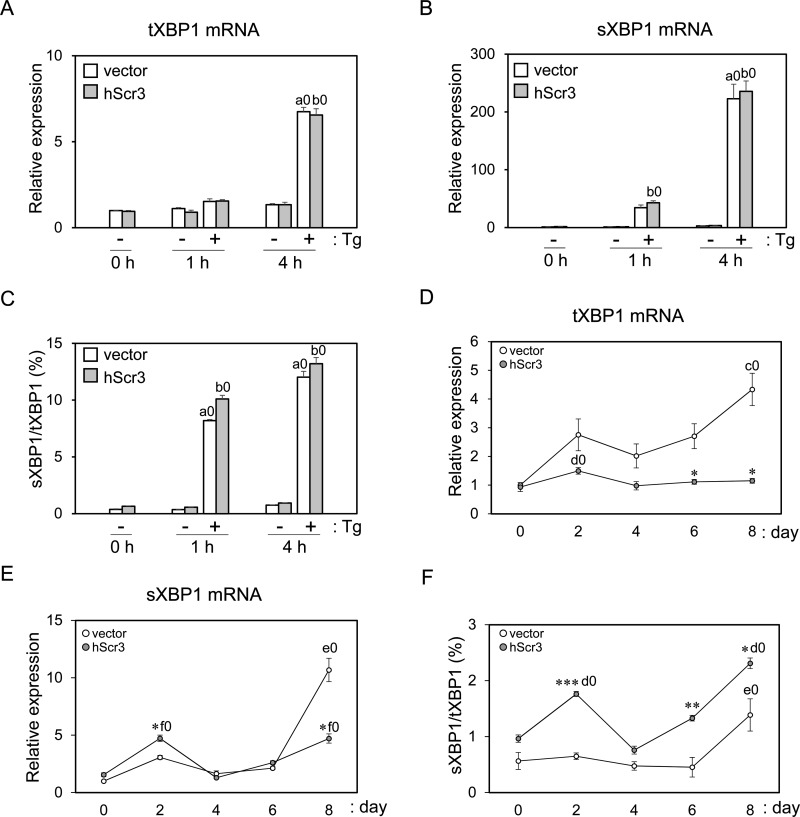
Effects of hScr3 overexpression on expression of ER stress-responsive XBP1 mRNA Control retrovirus-infected (unfilled columns) and hScr3-overexpressed 3T3-L1 cells (grey columns) in triplicate were treated with 2.5 nM Tg for 1 h or 4 h and subjected to RT-qPCR analysis of the expression of total (unspliced and spliced) XBP1 (tXBP1) mRNA (**A**) and spliced XBP1 (sXBP1) mRNA (**B**). Relative values compared with control cells on day 0 (set to 1.0) are shown in graph as mean ± S.E.M. (*n*=3). Ratio of sXBP1 mRNA to tXBP1 mRNA was calculated and is shown in (**C**). RT-qPCR analysis was also performed to investigate the expression pattern of tXBP1 mRNA (**D**) and sXBP1mRNA (**E**) during the course of differentiation of 3T3-L1 cells, and relative values compared with control cells on day 0 (set to 1.0) are shown in graph as mean ± S.E.M. (*n*=3). Unfilled circles, control cells; grey circles, hScr3-overexpressed cells. Ratio of sXBP1/tXBP1 mRNA is shown in (**F**). Statistical significance by Student's *t* test (*n*=3): *P<0.05; **P<0.01; ***P<0.001, hScr3-overexpressed cells compared with control cells*.* Statistical significance by Tukey's test (*n*=3): a0 (*P*<0.001), Tg treatment for 4 h (panels A and B) or 1 h and 4 h (panel C) compared with that for 0 h in control cells; b0 (*P*<0.001), Tg treatment for 4 h (panel A) or 1 h and 4 h (panels B and C) compared with that for 0 h in hScr3-overexpressed cells; c0 (*P*<0.01), day 8 compared with day 0 in control cells; d0 (*P*<0.05), day 2 (panel D) or days 2 and 8 (panel F) compared with day 0 in hScr3-overexpressed cells; e0 (*P*<0.001), day 8 compared with day 0 in control cells; f0 (*P*<0.001), days 2 and 8 compared with day 0 in hScr3-overexpressed cells.

We investigated the effects of hScr3 overexpression on UPR during adipocyte differentiation by analysing the expression levels of different XBP1 mRNA species including tXBP1 mRNA (total XBP1 mRNA: unspliced and spliced XBP1 mRNA) and sXBP1 mRNA by RT-qPCR using primers common to unspliced and spliced XBP1 mRNAs and primers specific to sXBP1 mRNA. First, to assess the technical reliability of the XBP1 mRNA analysis by RT-qPCR, we treated the cells with Tg, an irreversible SERCA inhibitor that is known to induce UPR by depleting the ER Ca^2+^ store [49,50]. As shown in Figures 6A–6C, Tg treatment of 3T3-L1 cells for 4 h induced the expression of tXBP1 mRNA (6.7-fold) and sXBP1 mRNA (235-fold), and the sXBP1/tXBP1 ratio was elevated from 0.37 to 13.2%. An increase in sXBP1 mRNA (42-fold) was also observed for 1-h Tg treatment, but tXBP1 mRNA induction was not evident. hScr3 did not affect the expression pattern of XBP1 mRNA and splicing in Tg-treated acute ER stress. Next, we analysed XBP1 mRNA during the course of adipocyte differentiation. The amounts of tXBP1 mRNA (Figure 6D) and sXBP1 mRNA (Figure 6E) in the control cells (empty vector retrovirus-infected 3T3-L1 cells, *unfilled circles*) have a statistically significant increase on day 8 compared with those on day 0. Although the degrees of increments were different between tXBP1 and sXBP1 mRNAs (5-fold and 11-fold on day 8 respectively), the tendency of expression was similar between the two mRNAs. Overexpression of hScr3 (grey circles) significantly suppressed the induction of tXBP1 mRNA (to 27% of the control) and reduced the sXBP1 mRNA level (43% of the control) on day 8. The ratios of sXBP1/tXBP1 ranged from 0.6 to 1.5% in control cells and 1 to 2.3% in hScr3-overexpressed cells (Figure 6F). Although Sha et al. [34] reported increase in total XBP1 mRNA similar to the one that we observed in control virus infected cells during adipocyte differentiation (Figure 6D), they showed that ratio of sXBP1 to tXBP1 mRNA was highest on day 0 (∼55% sXBP1). The substantial difference in the estimated content of sXBP1 mRNA (∼55% compared with 0.6%) may be partly explained by differences in the employed quantification methods (conventional RT-PCR followed by gel electrophoresis compared with RT-qPCR in the present study). Moreover, their culture condition may somehow activate IRE1α on day 0, but our culture condition may keep the cells unstressed.

## DISCUSSION

**Figure 7:**
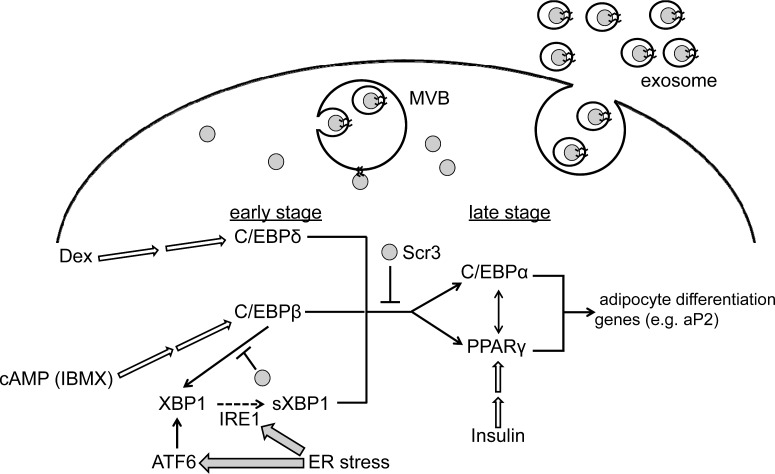
Working hypothesis for the negative regulatory function of Scr3 in 3T3-L1 adipogenesis Adipogenesis of 3T3-L1 cells is induced by glucocorticoids (Dex), cAMP (IBMX) and insulin, which induce or activate transcription factors (unfilled arrows). Thick solid lines with arrowheads indicate up-regulation of gene expression. ER stress-activated IRE1 triggers unconventional cytoplasmic splicing of XBP1 mRNA encoding an inactive transcription factor to generate sXBP1 mRNA encoding an active transcription factor, which also induces C/EBPα and PPARγ. Scr3 suppresses a transcriptional cascade leading to the expression of late stage pro-adipogenic transcription factors (C/EBPα and PPARγ) and XBP1 but has no effects on expression of early stage transcription factors (C/EBPβ and C/EBPδ). Scr3 may also suppress induction of XBP1 by C/EBPβ or ATF6 (transcription factor proteolytically activated in the Golgi apparatus by ER stress) (not shown). The Scr3 proteins, which are palmitoylated and internalized in intraluminal vesicles in multivesicular bodies (MVB), are released into the culture medium in the form of extracellular microvesicles (exosomes). Reduction in the amount of intracellular Scr3 protein enhances the adipogenic transcription factor cascade.

Adipocyte differentiation is regulated by a complex network of pro-adipogenic and anti-adipogenic cascades that are governed by signalling molecules from numerous pathways [42,43]. In the present study, we found that Scr3 functions as a negative regulator in adipogenesis of 3T3-L1 cells. Overexpression of hScr3 suppressed induction of late differentiation stage pro-adipogenic transcription factors (C/EBPα and PPARγ) but not early stage pro-adipogenic transcription factors (C/EBPβ and C/EBPδ) (Figure 5). Expression of C/EBPα and PPARγ is induced by mutual transacting activities of C/EBPα and PPARγ and by upstream activators including C/EBPβ, C/EBPδ and ER stress-activated spliced XBP1 (sXBP1) [34,43,51]. The mRNA of XBP1 including the cytoplasmically-unspliced inactive form is induced by C/EBPβ and ER stress-response ATF6 as well by sXBP1 as self-induction [34,52,53]. Scr3 may inhibit the cascade leading to transcriptional activation of the C/EBPα and PPARγ genes by inhibition of (i) both C/EBPβ and C/EBPδ, (ii) C/EBPβ alone, (iii) sXBP1, or (iv) all early stage transcription factors (see Figure 7 for working hypothesis). The possibility of additional suppression of the late stage transcription factors cannot be excluded. Interestingly, hScr3 overexpression inhibited induction of total (unspliced and spliced) XBP1 mRNA during the period of adipocyte differentiation (days 6 and 8) but not that of spliced XBP1 mRNA except for on day 8 (Figures 6D and 6E). Since hScr3 did not inhibit the expression of XBP1 mRNA or splicing in Tg-treated acute ER stress (Figures 6A–6C), Scr3 may have an effect, if any, on ER stress-response gene expression only in a state of chronic stress.

The molecular mechanism of the inhibitory function of Scr3 in adipogenesis is not known. In analogy of Scr1 function in the nucleus [27,28], Scr3 might act as a DNA-binding repressor or co-repressor of specific genes. Recent studies have indicated that Scr1 functions also as a negative regulator of virus-derived transacting factors such as HTLV-1 Tax, HIV Tat and Hepatitis B Virus X protein by physical interactions [54–56]. We presume that overexpressed Scr3 influences activities of certain transcription factors through direct or indirect interactions. To explore this possibility, studies are in progress to establish a luciferase-reporter assay system to investigate effects of Scr3 on transcriptional activities of C/EBPs using appropriate C/EBP-responsive gene promoters.

A different mechanism may underlie the slight enhancement of C/EBPβ induction and XBP1 mRNA splicing on day 2 by hScr3 overexpression (Figures 5A and 6E). C/EBPβ is induced by phosphorylated CREBP (cAMP response element-binding protein), which is initiated by IBMX (a phosphodiesterase inhibitor) included in the induction medium for the first 2 days [43]. It remains unknown whether cAMP also changes the phosphorylation state of Scr3, which is known to be phosphorylated by PKCδ or other unknown kinases [3,57], and regulates its function.

Decrease in Scr3 in the protein level (to 13.9–22.6%) more than in the mRNA level (to 33.2–54.0%) during adipocyte differentiation is partly explained by enhanced secretion of Scr3 in the form of extracellular microvesicles named exosomes (Figures 1–3 and Supplementary Figure S2). We previously reported that exosomal secretion of exogenously expressed Scr3 was significantly reduced and that subcellular localization was changed from the cytoplasmic puncta to nucleus by inhibition of palmitoylation of Scr3 by 2-bromopalmitate in HEK293 cells [18]. Since 2-bromopalmitate was toxic to 3T3-L1 cells when the cells were cultured for a long time, we could not investigate the effects of inhibition of palmitoylation of Scr3 on adipogenesis. Enhanced exosomal secretion during adipogenesis may not be specific to Scr3. Secretion of ALIX, which is an accessory protein of endosomal sorting complex required for transport (ESCRT) and an exosome marker [58], also increased (results not shown). Detailed biochemical studies are required in the future to determine whether palmitoylation of Scr3 and exosome secretion efficiency are regulated during adipogenesis. Exosomes containing a variety of proteins, miRNAs and lipids are known to function as intercellular communicators by acting on neighbouring or remote cells [58]. It would be interesting to determine whether enhanced secretion of Scr3 from adipocytes has biological significance in cell-to-cell communications in addition to decreasing the intracellular amounts of the protein. Alterations in efficiencies of synthesis (reduction) or degradation (enhancement) of Scr3 protein during differentiation may also partly explain the reduction in the amount of Scr3 protein in 3T3-L1 cells.

The adipogenic 3T3-L1 cell-line was derived from 3T3 Swiss albino fibroblasts [31–33]. Adipogenic stimuli caused a substantial enhancement in the induction of C/EBPα and PPARγ in 3T3-L1 cells but not in 3T3 cells as shown in Supplementary Figure S4, results that are consistent with results of previous studies by others [59]. Interestingly, although Scr3 was similarly expressed in 3T3 cells and 3T3-L1 cells under unstimulated conditions and its expression was transiently decreased on day 2, conspicuous differences in Scr3 expression levels were found between the two cell lines by continual adipogenic stimulations at the protein level (Figure 2) and at the mRNA level (Figure 3): (i) recovery to the unstimulated level in 3T3 cells but sustained at a low level in 3T3-L1 cells and (ii) a significantly higher rate of exosomal secretion of Scr3 protein in 3T3-L1 cells than in 3T3 cells. It remains unknown what genetic changes were generated during the conversion of 3T3 fibroblasts to adipogenesis-competent 3T3-L1 cells. Since forced overexpression of hScr3 caused suppression of adipogenesis, decrease in the amount of Scr3 protein in the cell is not a mere effect of adipogenesis but is likely to be an essential step for the differentiation. We attempted to knockdown Scr3 in 3T3-L1 cells and investigate whether this treatment accelerates the differentiation of adipocytes. Among the four purchased siRNAs, two of them were found to be effective for reducing expression of Scr3 mRNA but exhibited opposite effects on triacylglycerol accumulation (augmentation or suppression), most likely due to off-target effects. Although complementary supporting data are not yet available, this is the first report showing that Scr3 functions as a negative regulator in an adipogenic transcription cascade at least in part under the overexpressed condition. Future studies are required to determine whether this suppressive function of Scr3 is specific to adipogenesis of mouse 3T3-L1 cells or whether Scr3 also has a suppressive function in other murine and human cells.

## Abbreviations

aP2: adipocyte protein 2
C/EBP: CCAAT/enhancer-binding protein
Dex: dexamethasone
ER: endoplasmic reticulum
HEK: human embryonic kidney
hScr3 and mScr3: human and mouse phospholipid scramblase 3s (PLSCR3s)
IBMX: 3-isobutyl-1-methylxanthine
PPAR: peroxisome proliferator-activated receptor
RT-qPCR: reverse-transcription and real-time quantitative PCR
TCL: total cell lysate
Tg: thapsigargin
TUB: Tubby
TULP: Tubby-like protein
UPR: unfolded protein response
WB: Western blotting
XBP1: X-box-binding protein 1
